# Intramyocardial hemorrhage contributes to microvascular obstruction in acute myocardial infarction

**DOI:** 10.1186/1532-429X-14-S1-P19

**Published:** 2012-02-01

**Authors:** Nilesh R Ghugre, Jennifer Barry, Alan Moody, Bradley H Strauss, Graham Wright

**Affiliations:** 1 Imaging Research, Sunnybrook Research Institute, Toronto, ON, Canada; 2 Department of Medical Imaging, Sunnybrook Health Sciences Centre, Toronto, ON, Canada; 3 Schulich Heart Program, Sunnybrook Health Sciences Centre, Toronto, ON, Canada; 4 Department of Medical Biophysics, University of Toronto, Toronto, ON, Canada

## Summary

The clinical implications of hemorrhagic versus non-hemorrhagic infarcts are currently unclear. Our study suggests that hemorrhage may not simply be a bystander but an active contributor to adverse left-ventricular remodeling following acute myocardial infarction.

## Background

Patients with hemorrhagic infarcts appear to constitute a high-risk group in acute myocardial infarction (AMI). However, the clinical implications of hemorrhagic versus non-hemorrhagic infarcts are currently unclear, warranting a more systematic and mechanistic approach towards understanding the underlying consequences. The question of whether hemorrhage is simply a bystander or contributes to additional myocardial injury remains to be investigated. The purpose of the study was to artificially induce hemorrhage in normal and infarcted (but not hemorrhagic) porcine myocardium to determine whether hemorrhage, per se, worsens prior ischemic damage.

## Methods

Firstly, hemorrhage was induced in normal porcine hearts (N=18) by direct intracoronary injection of collagenase using over-the-wire angioplasty balloon catheter advanced to mid LAD after 2nd diagonal branch; balloon inflation was maintained for 8 min (ischemia). Six doses of (250,600,800,1200,1600,3200) mcg were administered in equally divided groups. Animals were sacrificed at 24 hrs and hearts were explanted for histological analysis. Secondly, hemorrhage was artificially induced in one animal subjected to a 45 min LAD occlusion. Collagenase was injected immediately after balloon deflation i.e. during reperfusion at an intermediate dose of 1000 mcg. For reference, another animal underwent a routine 45 min LAD occlusion. A comprehensive CMR examination was performed at day 2 post-AMI. Edema and hemorrhage were evaluated using T2 and T2* quantification, respectively, and infarction was assessed by delayed hyperenhancement (DHE) imaging.

## Results

**Figure 1:**
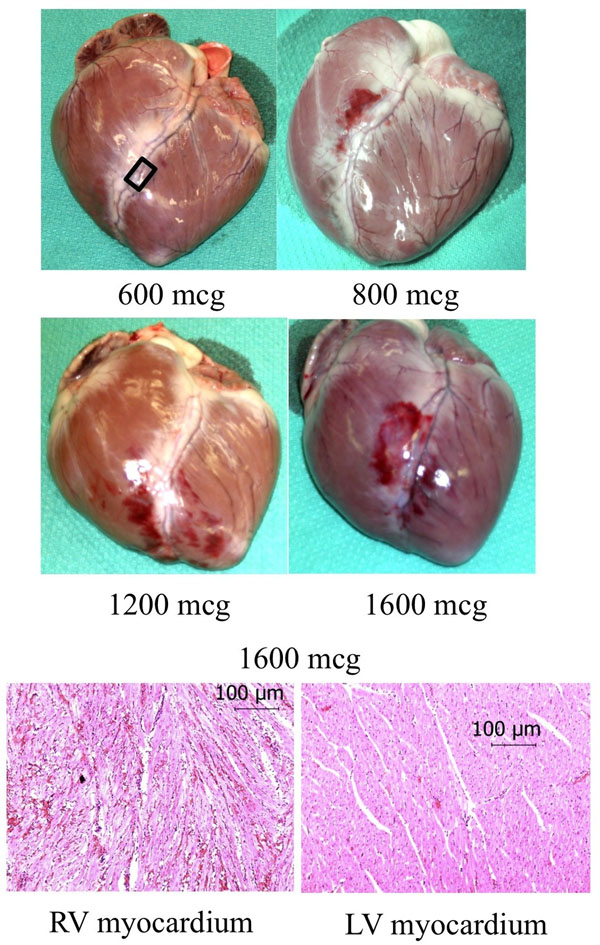
1st and 2nd row: Pig hearts treated with an intracoronary injection of collagenase beyond the second diagonal branch of the LAD (inset in 600 mcg image) after a brief ischemic episode of 8 min. For a dose of >800 mcg, collagenase resulted in hemorrhage (reddish areas) as is apparent on the explanted hearts; amount of hemorrhage increased with dose. 3rd row: Hematoxylin and eosin stains from the right and left ventricle (RV, LV) demonstrated widespread areas of red blood cells dispersed throughout the myocardium.

**Figure 2:**
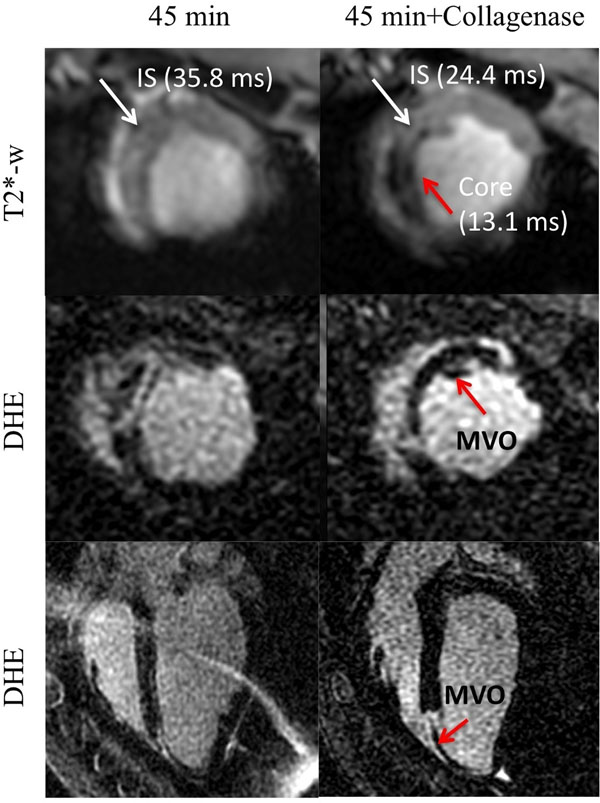
Representative short- and long-axis views from animals subjected to 45 min LAD occlusion without (left panel) and with collagenase (right panel) injection at day 2 post-AMI showing T2*-weighted (TE=15 ms) and delayed hyperenhancement (DHE) images. The 45 min infarction was non-hemorrhagic, non-transmural and heterogeneous. On the other hand, the collagenase-treated animal demonstrated a signal void on T2* image indicative of hemorrhage (red arrow) in association with a transmural infarction and microvascular obstruction (MVO, red arrow on DHE). This finding suggests an interaction between hemorrhage, MVO and infarction.

In the control animals, there was no mortality attributable to collagenase infusion. Epicardial and intramyocardial hemorrhage was observed in a dose-dependent manner with none or mild, focal hemorrhage up to 600 mcg, mild-moderate at 800-1600 mcg and severe at 3200 mcg (Fig. 1); no infarction was observed. In the collagenase treated infarction (Fig. 2), MRI examination at day 2 post-AMI revealed signal void on T2*-weighted images, indicative of hemorrhage. Alongside a surprising yet interesting finding was the presence of microvascular obstruction (MVO) on DHE images. This was unlike the reference 45 min infarction, which was non-hemorrhagic and with no MVO.

## Conclusions

Hemorrhage has always been found to be associated with MVO, however, the causal relationship between the two is currently unknown. We speculate that blood spilt in the interstitium might have compressed the microvasculature that was already vulnerable due to the initial ischemic insult; in other words, hemorrhage may have created the MVO. Our preliminary study suggests that hemorrhage may not simply be a bystander but an active contributor to adverse left-ventricular remodeling following AMI.

## Funding

We would like to acknowledge funding support from the Ontario Research Fund, the Canadian Institutes of Health Research and GE Healthcare.

